# Time trends in stomach cancer mortality across the BRICS: an age-period-cohort analysis for the GBD 2021

**DOI:** 10.3389/fpubh.2025.1506925

**Published:** 2025-02-28

**Authors:** Dan Liu, Hao Liu, Yuhang Wu, Weihong Wang

**Affiliations:** ^1^Medical College of Hunan Normal University, Changsha, China; ^2^Prehospital Emergency Department of Xiangtan Central Hospital, Xiangtan, China; ^3^State Key Laboratory of Natural Medicines, Key Laboratory of Drug Metabolism, China Pharmaceutical University, Nanjing, China; ^4^Department of Epidemiology and Health Statistics, Xiangya School of Public Health, Central South University, Changsha, China

**Keywords:** stomach cancer, mortality, age-period-cohort model, BRICS, GBD analysis

## Abstract

**Objectives:**

Stomach cancer is one of the leading causes of cancer death, and its epidemiologic characteristics are regionally heterogeneous worldwide. The BRICS nations (Brazil, Russian Federation, India, China, and South Africa) have markedly increasing influences on the international stage. We aim to investigate time trends in stomach cancer mortality among the BRICS countries from 1982 to 2021.

**Methods:**

Data for this study were obtained from the Global Burden of Disease (GBD) 2021 public dataset to investigate the deaths, all-age mortality rate, and age-standardized mortality rate (ASMR) of stomach cancer. The age-period-cohort (APC) model was employed to estimate net drift, local drift, age-specific curves, and period (cohort) relative risks, and the Bayesian generalized linear model was employed to evaluate the relationship between food intake and mortality rate.

**Results:**

In 2021, there were approximately 572,000 stomach cancer deaths across the BRICS, accounting for 59.9% of global death. Russian Federation exhibited the most significant reduction in ASMR of stomach cancer among the BRICS. In contrast, China continued to report the highest number of stomach cancer deaths. The risk of mortality associated with stomach cancer exhibited a marked increase with advancing age, both within these countries and at the global level. PUFA, sodium, calcium and trans fat may have an impact on the mortality rate of stomach cancer. Favorable trends in period and birth cohort effects were observed in these five nations over the past decades.

**Conclusion:**

BRICS countries have made varying progress in reducing stomach cancer mortality. Given the diverse environments, it is recommended to progressively develop customized stomach cancer prevention strategies, utilizing available resources. Healthcare services should be extended to all age groups, with a particular emphasis on vulnerable populations.

## Introduction

Stomach cancer is characterized as an aggressive malignancy (1) and typically manifests with a range of symptoms, including abdominal pain, gastric outlet obstruction, and hemorrhage at the primary tumor site, all of which substantially impair the quality of life for affected patients (2). In 2022, it accounted for 968,350 new cases and 659,853 deaths, ranking fifth in terms of incidence and mortality among cancers worldwide (3). This situation results in a substantial burden on individuals and healthcare systems, particularly in low- and middle-income countries. In 2015, the United Nations (UN) set a target to reduce premature mortality from non-communicable diseases by one-third by the year 2030, including cancers (4). The BRICS nations play a crucial role in achieving this UN target.

The BRICS countries (including Brazil, Russian Federation, India, China, and South Africa) are characterized by rapid economic growth and have formed a powerful political and economic alliance, collectively responsible for nearly half of the global population (5). The importance of studying stomach cancer in these nations lies in their large populations and the substantial health burden this disease may pose (6–8). Considering these factors, investigating the status of stomach cancer in BRICS countries is crucial for understanding its epidemiology, including risk factors and potential prevention and management strategies. The Global Burden of Disease (GBD) 2021 offers a revised and comprehensive dataset, which has become a key to analyzing stomach cancer mortality trends within these countries (9). Several studies have analyzed stomach cancer mortality over time, reporting changes in age-specific mortality rates at a global level (10–12). However, these studies fail to identify specific risk population and the disease’s etiological factors, which are essential for assessing the effectiveness of prior policy interventions and identifying future targets. The Age-Period-Cohort (APC) model is a sophisticated analytical framework that is particularly adept at unraveling the intricate relationships between age, period, and cohort effects on stomach cancer mortality (13). By applying this model, researchers can identify critical periods for intervention and assess the effects of generational differences, thereby enhancing the precision of public health strategies. Furthermore, due to the significant dietary disparities across BRICS countries, we further modeled the potential associations between dietary components and stomach cancer mortality, aiming to deepen the understanding of the underlying factors contributing to stomach cancer mortality in these nations.

The present study employs the latest data from the GBD 2021 to investigate temporal trends in stomach cancer mortality and the relative proportions of mortality across various age groups. Furthermore, we examine variations in mortality trends by age, period, and birth cohort among the BRICS countries from 1982 to 2021.

## Method

### Data sources

This study utilized data from the GBD 2021 database, accessible via the Global Health Data Exchange GBD Results Tool.^1^ GBD 2021 offers up-to-date and comprehensive insights into the burden of 371 diseases in 204 countries and territories worldwide (9). This repository contains a wealth of data on disease burden, risks, mortality, and disability, making it a crucial resource for understanding global public health. Furthermore, GBD 2021 considers the impact of the COVID-19 pandemic on the global disease burden (9).

In the GBD 2021, stomach cancer is defined according to the International Classification of Diseases, 10th edition (ICD-10), as C16.0–16.9 (14). We gathered data on the numbers of stomach cancer patients, deaths among males and females, as well as age-specific mortality rates and age-standardized mortality rates (ASMR) for individuals aged 15 to 94 years in both global and BRICS countries from 1982 to 2021. Ninety-five percent uncertainty intervals (95% UI) for each metric were provided based on the 2.5th and 97.5th ordered values from 1,000 draws from the posterior distribution (9). Additional details on the specific data, methodologies utilized, and statistical modeling for GBD 2021 are available in other sources (9, 15, 16). The data were anonymized and made publicly accessible, with the informed consent waiver reviewed and approved by the University of Washington Institutional Review Board.

The data regarding the dietary intake of 15 food items were sourced from the GBD 2021 Dietary Risk Exposure Estimates spanning from 1990 to 2021. This dataset contains detailed information on the daily per capita consumption, measured either in grams per day or energy per day, segmented by country, age, and sex. The choice of these particular 15 dietary elements was made in strict accordance with the criteria set forth by the Global Burden of Disease Study for the selection of risk factors. The food items under analysis comprised milk, nuts, omega-3 fatty acids, polyunsaturated fatty acids (PUFA), dietary sodium, red meat, trans fats, vegetables, legumes, calcium, sugar-sweetened beverages (SSBS), processed meat, fruits, and dietary fiber. All forms of relevant dietary data were incorporated into the analytical process (17).

### Statistical analysis

#### Age-period-cohort modeling analysis

In this study, the APC model is utilized to analyze the mortality data, with age, period, and cohort serving as independent variables. The model considers the incidence rate of observed events within the population as the dependent variable, based on a specific probability distribution (18). By employing the APC methodology, this study transcends traditional epidemiological approaches to elucidate the impact of various factors on disease dynamics as well. Within the APC model, the results offer several crucial indicators: net drift, local drift, longitudinal age curve, period relative risk (RR), and cohort relative risk. Net drift encapsulates the comprehensive log-linear trend of mortality rates across the entire population, taking into account both period and cohort variations. Local drift indicates the trend within each specific age bracket. The longitudinal age curve illustrates the expected age-specific rates for a reference cohort, with adjustments made for period effects. Period rate ratios (RR) and cohort RRs assess relative risks across different periods and cohorts, respectively, making adjustments for age as well as the comparative factor (either period or cohort) (13). Age, period, and cohort effects were analyzed using Poisson regression, formulated as follows:


$$g\;\left(\frac{Y_{j}}{\mu }\right)=\log\;\left(\lambda_{j}\right)=u+\alpha *\mathrm{ag}e_{j}+\beta *\mathrm{perio}d_{j}+\gamma *\mathrm{cohor}t_{j}$$

In this equation, $\lambda_{j}$ denotes the response variable representing the net effect on the mortality of stomach cancer for group j , while $Y_{j}$ and μ indicate the number of mortality and the population at risk, respectively. The coefficients α , β , and γ correspond to the effects of age, period, and birth cohort within the APC model, respectively, and *u* represents the model’s intercept. To address the non-identification issue inherent in the APC model, we utilized the Intrinsic Estimator (IE) method, which is recognized for providing superior fit compared to alternative modeling approaches (19).

#### Bayesian generalized linear modeling analysis

In accordance with Bayesian theory, a Bayesian generalized linear model was constructed through the Markov Chain Monte Carlo (MCMC) approach. This model sought to investigate the association between the incidence of stomach cancer and the daily per capita consumption of 15 food items. By identifying significant variables, their intake proportions in different countries were examined, enabling a thorough exploration of the relationship between incidence rates and dietary intake disparities across diverse environments. In this model, the observed data pertained to stomach cancer mortality rates, and the explanatory variables were the per capita intakes of the 15 food items. The fixed effects within the model encompassed the influence of food intake on stomach cancer incidence, while year, age, and sex were integrated as random effects.

#### Data arrangement

The APC model necessitates that the age group and period group data be consistently structured to ensure robust and valid analytical outcomes. Therefore, we categorized stomach cancer mortality and population data according to predefined criteria. Ages were segmented into 5-year intervals (15–19, 20–24, …, 90–94 years). Age groups younger than 15 years and older than 94 years were excluded due to the rarity or absence of stomach cancer deaths. GBD data were harmonized into a unified framework by extracting death and population counts at eight specific mid-year time points (1984, 1989, …, 2019) rather than using five-year averages for each period. Birth cohorts, defined by subjects’ ages and event dates (cohort = period − age), ranged from 1890–1894 (median year 1892) to 2000–2004 (median year 2002). Estimated parameters were acquired through APC analyses using the age-period-cohort web tool developed by the National Cancer Institute and visualized with the R statistical program (version 4.2.3). The significance of assessable parameters and functions was examined using the Wald $\chi^{2}$ test, with all statistical tests conducted as two-sided.

## Results

### Death of stomach cancer trends from 1982 to 2021

**Figure 1:**
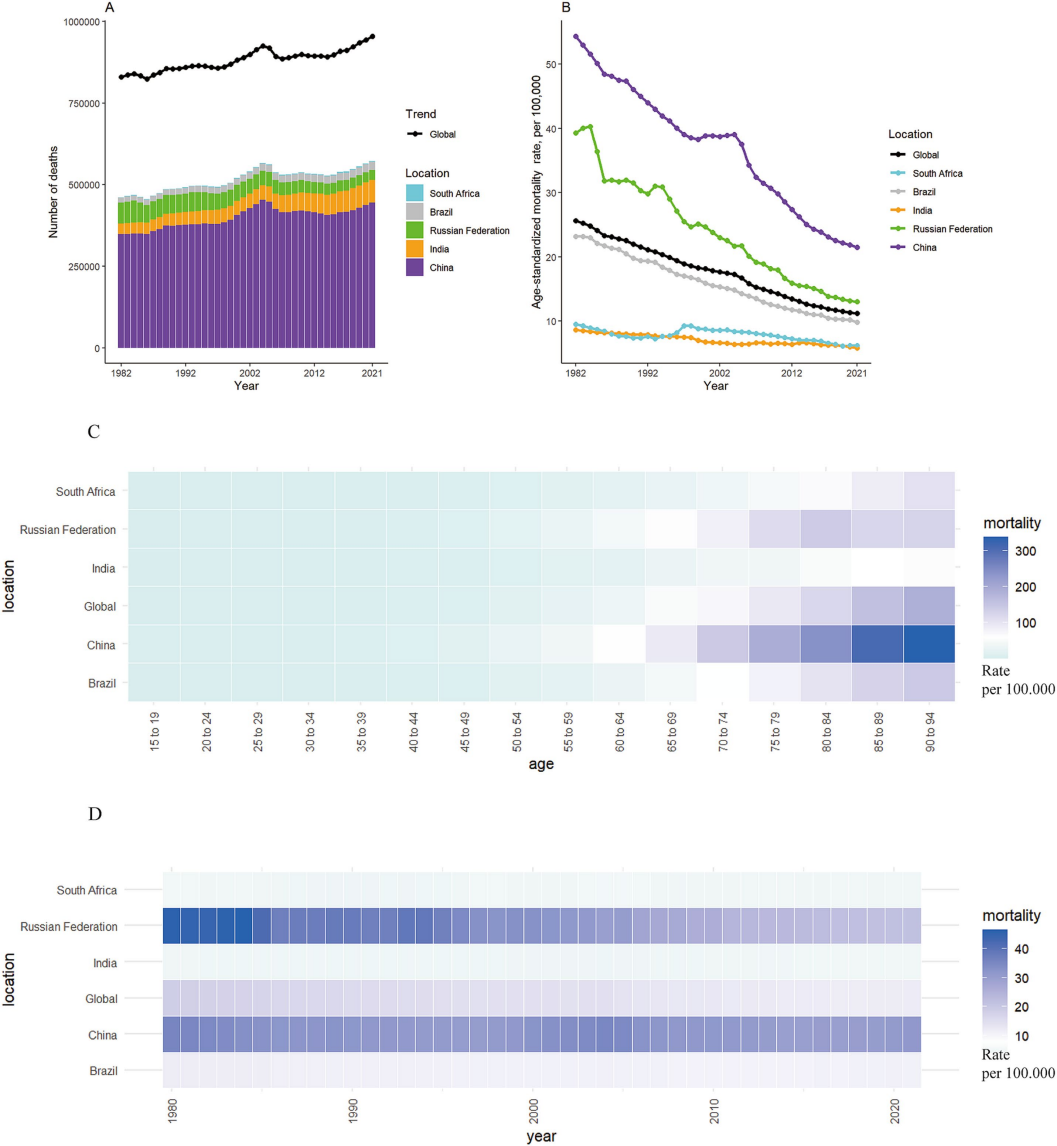
The mortality of stomach cancer in global and BRICS from 1982 to 2021. **(A,B)** The number and age-standardized rate of stomach cancer mortality worldwide and in BRICS countries from 1982 to 2021. **(C)** Heatmap of stomach cancer mortality rates by age in BRICS countries in 2021. **(D)** Heatmap of stomach cancer mortality rates in BRICS countries from 1982 to 2021.

Table 1 presents the population, total number of stomach cancer deaths, all-age mortality rate, age-standardized mortality rate, and net drift of mortality. Between 1982 and 2021, there was a 15.03% increase in stomach cancer deaths worldwide, rising from about 830,000 (95% UI 752,000 to 905,000) to 954,000 (95% UI 822,000 to 1,090,000) (Figure 1A). The ASMR decreased from 25.64 per 100,000 (95% UI 23.34 to 27.81) to 11.20 per 100,000 (95% UI 9.62 to 12.73), reflecting a 56.32% reduction (Figure 1B). The APC model estimated a net drift of −2.53% (95% confidence interval [CI], −2.60 to −2.47) in the stomach cancer mortality rate from 1982 to 2021 globally (Table 1).

**Table 1:** Trends in stomach cancer mortality across BRICS, 1982–2021.

	Global		Brazil		Russian Federation		India		China		South Africa	
	1982	2021	1982	2021	1982	2021	1982	2021	1982	2021	1982	2021
Population												
Number, *n* × 1,000,000	4,634 (4,542, 4,371)	7,891 (7,668, 8,131)	129 (118, 140)	220 (188, 251)	143 (131, 155)	145 (125, 164)	728 (673, 787)	1,415 (1,240, 1,602)	1,006 (938, 1,073)	1,423 (1,319, 1,530)	31 (26, 35)	57 (50, 64)
Percentage of global, %	100	100	2.8	2.8	3.1	1.8	15.7	17.9	21.7	18	0.7	0.7
Deaths												
Number, *n* × 1,000	830 (752, 905)	954 (822, 1,090)	15 (14, 16)	24 (23, 26)	64 (62, 65)	31 (29, 34)	33 (27, 42)	69 (59, 84)	348 (286, 413)	445 (345, 556)	2 (1, 2)	3 (2, 3)
Percentage of global, %	100	100	1.8	2.5	7.7	3.3	4	7.1	42	46.6	0.2	0.3
Percent change of mortality 1982–2021, %	15.03		62.37		−51.19		110.04		27.77		78.26	
All-age mortality rate												
Rate per 100,000	17.90 (16.24, 19.52)	12.09 (10.41, 13.81)	11.67 (11.12, 12.11)	11.06 (10.26, 11.65)	44.60 (43.40, 45.68)	21.49 (19.72,23.25)	4.48 (3.73, 5.77)	4.84 (4.20, 5.96)	34.62 (28.41, 41.04)	31.28 (24.23, 39.07)	5.01 (3.76, 5.58)	4.83 (4.11, 5.47)
Percent change of rate 1982–2021, %	−32.46		−5.23		−51.82		8.04		−9.65		−3.59	
Age-standardized mortality rate												
Rate per 100,000	25.64 (23.34, 27.81)	11.20 (9.62, 12.73)	23.18 (21.93, 24.18)	9.81 (9.07, 10.34)	39.30 (38.20, 40.30)	13.00 (11.94, 14.05)	8.65 (7.19, 11.30)	5.83 (5.06, 7.15)	54.32 (45.21, 63.97)	21.51 (16.66, 26.61)	9.53 (7.13, 10.07)	6.20 (5.18,6.97)
Percent change of rate 1982–2021, %	−56.32		−57.68		−66.92		−32.6		−60.4		−34.94	
APC model estimates												
Net drift of mortality rate, % per year	−2.53 (−2.60, −2.47)		−2.17 (−2.25, −2.10)		−3.31 (−3.56, −3.05)		−1.30 (−1.48, −1.12)		−2.76 (−2.90, −2.63)		−1.37 (−1.62, −1.13)	

All-age mortality = crude mortality rate. Age-standardized mortality rate is computed by direct standardization with global standard population in GBD 2021. Net drifts are estimates derived from the age-period-cohort model and denotes overall annual percentage change in mortality, which captures the contribution of the effects from calendar time and successive birth cohorts. Parentheses for all GBD health estimate indicate 95% uncertainty intervals; parentheses for net drift indicate 95% confidence intervals; APC, age-period-cohort.

The reduction of stomach cancer mortality was most marked for the Russian Federation, with a striking decrease in ASMR from 39.30 per 100,000 to 13.00 per 100,000 (changes: 66.92%) (Figures 1B–D). Despite notable reduction of 60.40% in ASMR for stomach cancer, China had the highest ASMR at 21.51 per 100,000 in 2021 among the BRICS nations. The reductions in mortality were less pronounced in Brazil, with a decrease of 57.68%. In contrast, the ASMR of stomach cancer fell slightly in India and South Africa, with declines of 32.60 and 34.94%, respectively. According to the APC model estimates, the annual net drift in stomach cancer mortality ranged from −3.31% (95% CI, −3.56 to −3.05) in Russian Federation to −1.30% (95% CI, −1.48 to −1.12) in India within the BRICS countries (Table 1).

### Time trends in stomach cancer mortality across different age groups

**Figure 2:**
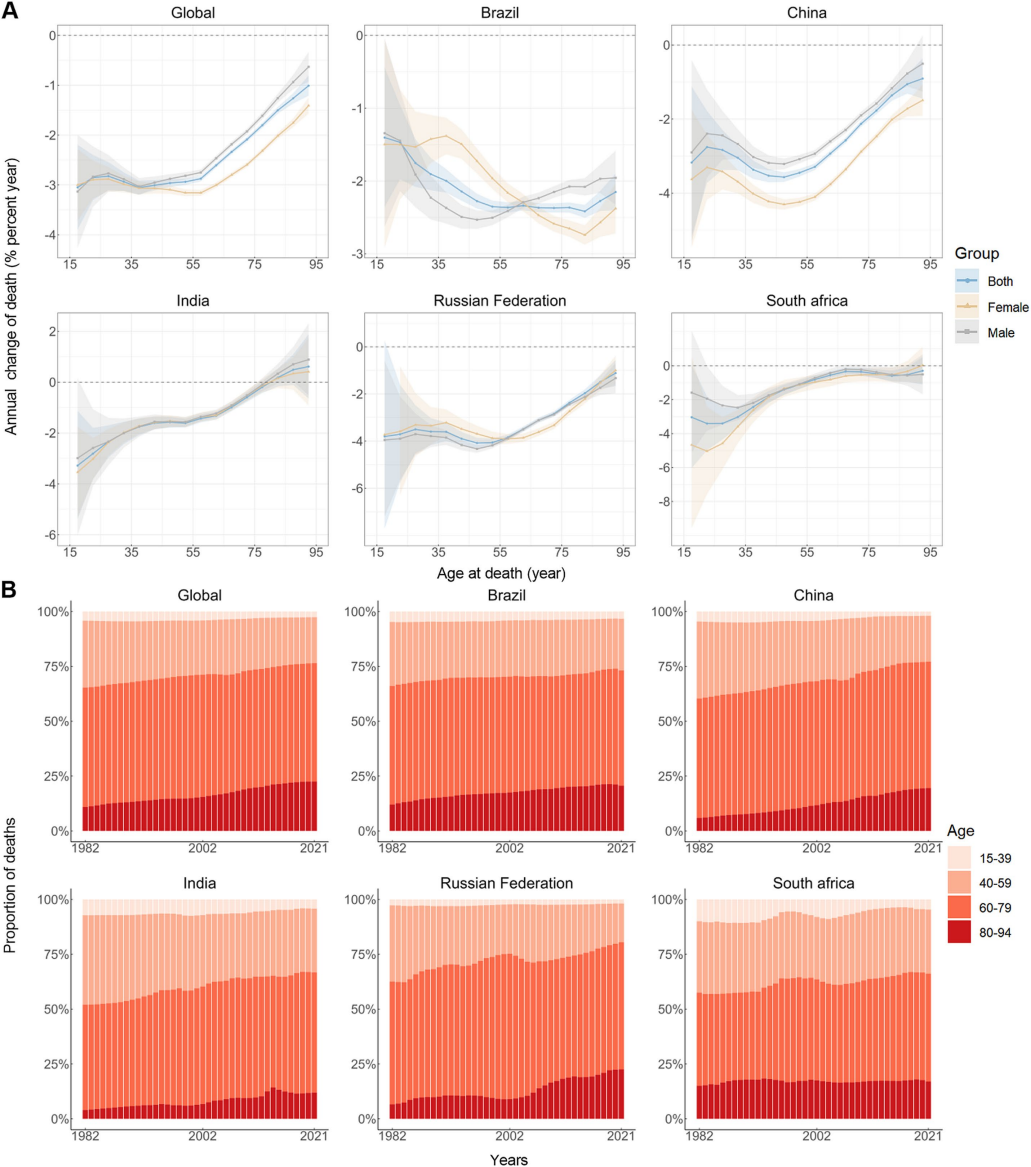
Local drifts of mortality rate and age distribution of deaths in global and BRICS, 1982–2021. **(A)** Local drifts of stomach cancer mortality rate (estimates from age-period-cohort models) for 16 age groups (15–19 to 90–94 years), 1982–2021. The dots and shaded areas indicate the annual percentage change of mortality rate (% per year) and the corresponding 95% CIs. **(B)** Temporal change in the relative proportion of stomach cancer deaths across age groups, 1982–2021.

The annual percentage change in stomach cancer mortality rates across different age groups is shown in Figure 2A. For the majority of age groups across both sexes, the values predominantly fall below zero, indicating reductions in stomach cancer mortality. Exceptions include both males and females aged 75–94 in India and females aged 90–94 in South Africa. Figure 2B illustrates the temporal evolution of the age distribution of stomach cancer mortality. Individuals aged 40 to 79 years constitute the majority of stomach cancer-related deaths. A notable trend is observed, where the mortality rate for stomach cancer consistently increases with advancing age across the BRICS (Figure 1C).

### Age, period and cohort effects on stomach cancer

An Age-Period-Cohort-Interaction Effects (APC-IE) analysis was conducted to disentangle the interactions among age, period, and cohort factors. We computed the coefficients for age, period, and birth cohort (Table 1) for mortality using the APC-IE framework. Subsequently, we calculated the relative risks of stomach cancer and mortality based on these coefficients to assess the impact of each individual factor on the risk of stomach cancer mortality.

**Figure 3:**
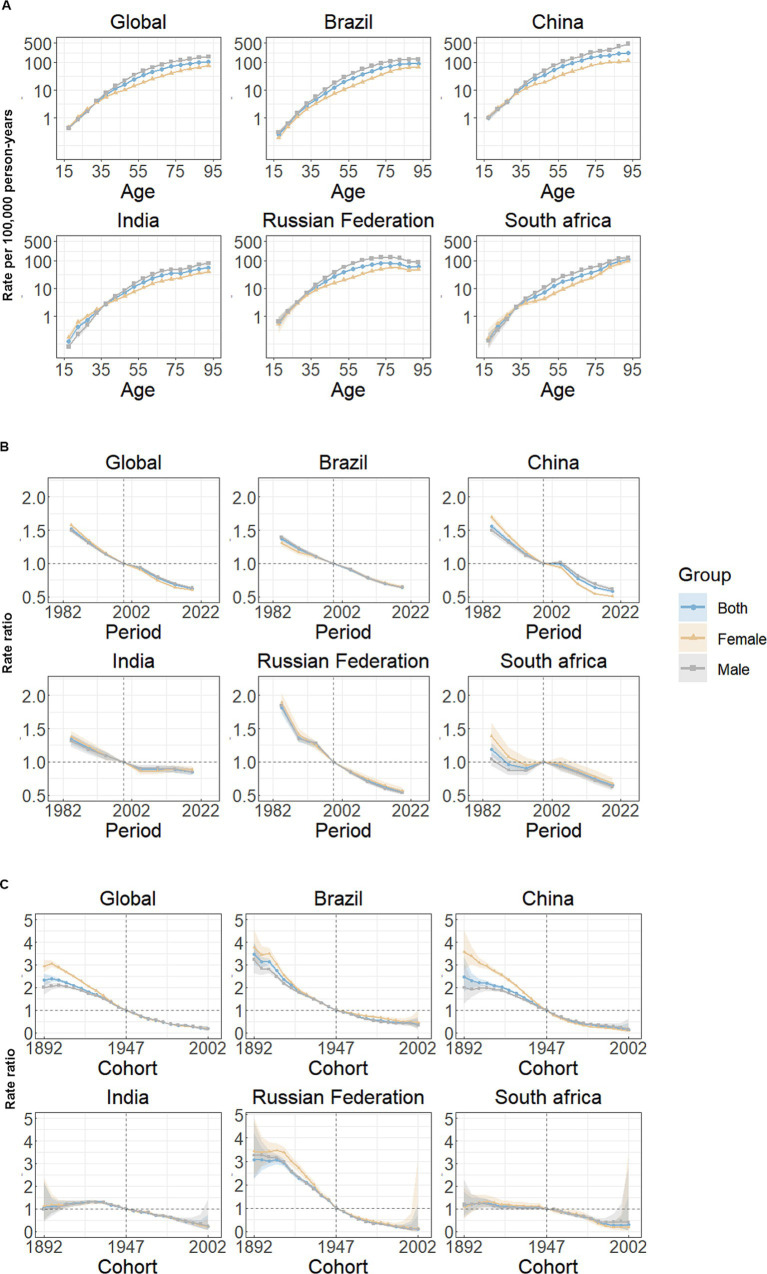
Age, period and cohort effects on stomach cancer mortality in global and BRICS. **(A)** Age effects are shown by the fitted longitudinal age curves of mortality rate (per 100,000 person-years) adjusted for period deviations. **(B)** Period effects are shown by the relative risk of mortality rate (mortality rate ratio) and computed as the ratio of age-specific rates from 1982–1986 to 2017–2021, with the referent period set at 1997–2001. **(C)** Cohort effects are shown by the relative risk of mortality rate and computed as the ratio of age-specific rates from the 1892 cohort to the 2002 cohort, with the referent cohort set at 1947. The dots and shaded areas denote mortality rates or rate ratios and their corresponding 95% CIs.

Figure 3 illustrates the estimates of age-period-cohort effects for both global and BRICS nations. The risk of mortality attributed to stomach cancer exhibits a marked increase with advancing age (Figure 3A). Among all study subjects, excluding the Russian Federation, the rates of mortality from stomach cancer were highest in the age group of 90 to 94 years for both women and men, individuals 80 to 84 years of age had the highest mortality rates in the Russian Federation (Figure 3A; Table 1). Marked gender disparities are present in mortality rates, with males experiencing notably higher rates than females for almost all age groups. Notably, in contrast, females exhibit higher mortality risks than males in the younger age group (15–34 years) in India. Similar trends were observed for both individual countries and globally, showing substantial improvements in mortality risk. The period effect indicated that mortality rates decreased from 1982 to 2021, irrespective of sex (Figure 3B; Table 1). When compared to 1999, the mortality rate was lowest in 2019 (0.62, 95% CI: 0.61, 0.63) and highest in 1984 (1.52, 95% CI: 1.49, 1.55) globally (Figure 3B; Table 1). In India, the period effects were relatively stable, indicating few improvements for the overall population throughout the study period (Figure 3B). Globally, there has been a continuous improvement in cohort effects across successive birth cohorts, a pattern similarly observed in all BRICS nations. Mortality risks were higher in the earlier birth cohort (before 1945) than in the centralized birth cohort (1945–1949) (Figure 3C; Table 1). Remarkably, Brazil, China, and Russian Federation had more favorable trends than the global average, while only small improvements were seen in India and South Africa (Figure 3C).

### The correlation between food composition and stomach cancer mortality rate

In this study, we employed a Bayesian Generalized Linear Model to examine the potential impact of dietary patterns on stomach cancer mortality in the BRICS nations. By comparing global dietary types with cancer mortality rates, we identified that four specific food components—PUFA (polyunsaturated fatty acids), Sodium, trans fat, and Calcium—played a significant role in influencing the variation in stomach cancer mortality rates in the BRICS countries. Specifically, the changes in these four components exhibited a remarkable effect, with absolute posterior mean (post.mean) values exceeding 40 (as shown in Table 2). These findings highlight the importance of closely monitoring the intake of these nutrients in BRICS nations, as their dietary patterns may have substantial implications for stomach cancer mortality rates.

**Table 2:** Intake of 15 different foods of BRICS in 2021 and the associated significance *p*-value with the mortality rate of stomach cancer.

Food type	Global	Brazil	China	India	Russian Federation	South Africa	BGLMM model *p* value	post.mean
Milk	95.5090592	184.472331	19.5403251	26.026126	249.845936	83.0174329	<0.001	0.4617
Nuts	9.01539768	23.0893284	7.16669885	3.66084033	3.94322022	2.4604687	<0.001	−10.2265
Omega-3	0.42836999	0.24622666	0.51430224	0.20527288	0.46404579	0.1925409	0.074	51.5579
PUFA	2.76700506	6.35624923	2.03595009	1.31641259	2.06511825	4.07214416	<0.001	46.0661
Sodium	4.3369167	3.53691892	6.82655106	3.72399056	3.82727554	2.42967846	<0.001	−46.002
Red meat	31.9611218	63.4101142	47.1956457	3.26795497	35.0409214	31.4793116	<0.001	−1.6537
Trans fat	0.47183038	0.57159578	0.42823759	0.81249474	0.33111748	0.2236267	<0.001	−233.7877 -
Vegetable	233.642075	106.038995	450.498811	111.376465	182.107457	91.1040866	<0.001	−0.7031
Whole grains	29.8754706	38.4814114	24.5564856	39.6660973	16.7659057	55.2734465	<0.001	5.1712
Legumes	37.5757135	96.1163119	45.68779	34.6410518	13.4316086	21.1828156	<0.001	1.0431
Calcium	0.57034992	0.66167344	0.48319073	0.4952821	0.81680414	0.30715495	<0.001	−345.9139
SSBS	44.9300551	66.3079481	19.5781714	27.5408552	35.916673	50.4489253	<0.001	1.9236
Processed meat	12.7615636	7.04865896	4.40132376	0.87047526	30.0187661	5.7645511	<0.001	0.799
Fruit	154.662068	250.405059	192.645003	17.7664244	132.386346	62.7268292	<0.001	1.6111
Fiber	18.3405469	17.4285224	20.4067552	18.0048862	17.8535423	21.0934901	<0.001	21.9075

## Discussion

In this study, we employed data from the GBD 2021 to comprehensively analyze the mortality burden attributable to stomach cancer within the BRICS nations. Encouragingly, there has been a discernible decline in the ASMR related to stomach cancer, both on a global scale and within the BRICS region. Significant disparities were observed among BRICS nations concerning both stomach cancer mortality rates and the temporal trends associated with these rates.

Between 1982 and 2021, the total number of stomach cancer deaths increased both in individual countries and globally, in part as a result of population growth. However, the ASMR for stomach cancer demonstrated an adverse trend over the 40-year period. The striking improvements were likely attributed to a deeper understanding and research into the mechanisms of stomach cancer onset, including factors such as *Helicobacter pylori*, miRNAs, and the application of endoscopic screening, early diagnosis and treatment (20–23). Additionally, the continuous updates of stomach cancer diagnosis and treatment guidelines by various countries and authoritative medical organizations were significant factors contributing to the favorable trend (24–26).

Although BRICS countries stand out due to their rapidly changing socio-economic landscapes and large populations, significant imbalances in their economic and social development persist, particularly in terms of regional disparities, urban–rural divides, and wealth inequality. These imbalances are also reflected in health outcomes shaped by distinct healthcare systems, environmental exposures, and public health policies. *H. pylori* infection has been identified as one of the major etiological factors for stomach cancer. While the prevalence of *H. pylori* infection has declined in recent decades, it remains highly prevalent in BRICS countries, contributing significantly to the disease burden of stomach cancer (27, 28). These nations have made notable efforts to address *H. pylori* infection, such as strong stomach cancer screening programs in certain regions of Russia and China, alongside the gradual implementation of treatment guidelines. Therapies, including proton pump inhibitors (PPIs) and antibiotics, have been used to treat *H. pylori* infections. However, the issue of antibiotic resistance has led to the abandonment of first-line treatments in China, particularly for patients with clarithromycin resistance. Additionally, in some areas of Russia, Brazil, and South Africa, disparities in healthcare resource distribution and socio-economic factors limit the coverage and effectiveness of treatment. In India, *H. pylori* treatment is still in the early stages, and progress in coverage has been slower compared to other BRICS countries, with widespread issues of antibiotic resistance remaining. The substantial wealth gap and insufficient basic healthcare infrastructure contribute to the imbalance in healthcare resources between urban and rural areas, leading to considerable differences in treatment outcomes. Furthermore, the distribution of deaths attributed to stomach cancer shows a transition from younger to older age groups, which may partly be due to the increasing utilization of screening and monitoring programs among younger individuals, resulting in the detection of cancers at earlier stages (29). Population aging is also likely a significant factor contributing to this shift (30).

The greatest decline in stomach cancer mortality was observed in The Russian Federation across the BRICS, with both period and cohort effects indicating generally favorable trends. It is probable that Russia’s healthcare policies were a significant contributing factor. The Russian Federation inherited the medical system from the former Soviet Union, which emphasized the importance of prevention and accessibility (31). The country prioritized cancer diagnosis and treatment as a healthcare focus area and subsequently introduced the “National Strategy for Oncology Treatment and Prevention,” significantly reducing stomach cancer mortality rates (31). Smoking control may be another important contributor. A comprehensive series of tobacco control measures was implemented by the Russian government including the ratification of the “Framework Convention on Tobacco Control” in 2008, followed by a succession of related policies (32). Notably, between 2009 and 2016, The Russian Federation witnessed a substantial 21.5% reduction in smoking prevalence (33).

Despite an increasing absolute number of stomach cancer deaths attributable to population aging, China demonstrated favorable temporal trends in ASMR related to stomach cancer, with improvements observed over time and across birth cohorts. China’s substantial investment in public health initiatives likely played a significant role in these improvements, including the establishment of China’s first nationally significant public health project for cancer prevention and control, the initiation of the stomach cancer early screening program (34), and the onset of “China Cancer Prevention and Control Plan (2004–2010) (35).” Furthermore, the substantial economic benefits resulting from China’s reform and opening-up policies have significantly improved living conditions, enhanced food storage practices, reduced the presence of carcinogenic substances in food, and enabled the ongoing enhancement of the national healthcare system by the Chinese government (36).

Brazil exhibited a marked reduction in stomach cancer mortality, with both period and cohort effects indicating generally favorable trends. Several factors likely contributed to this observed trend: the implementation of the Unified Health System (SUS) in 1988 and subsequent family health programs, which expanded access to healthcare services, diagnostic resources, and screening programs for underserved populations, government initiatives to incentivize healthcare professionals to practice in rural and remote areas; enhanced nationwide coverage; and continuous updates of the Mortality Information System (SIM) (37–39). Additionally, the reduction in the prevalence of *H. pylori* infection, along with the increased use of refrigerators—which has led to higher consumption of fruits and vegetables—and a decrease in the consumption of salt-preserved food (40) may be significant contributors.

Despite significant efforts by the governments, the mortality reductions were less striking in both India and South Africa. The observed trend indicated that over the past four decades, there had been limited improvements in the burden of stomach cancer. India is home to 1.39 billion people, with a rapid increase. Population growth and aging present formidable sustainability challenges to the financing of healthcare systems and the overall integrity and functionality of the health infrastructure (41). India’s Health systems, which have been insufficiently responsive to the escalating threat of noncommunicable diseases (5) may be another contributor to the trend. Lastly, factors such as low healthcare expenditure, inaccessible health services, high tobacco usage rates, and severe shortage of healthcare professionals probably play a significant role in the limited improvements in stomach cancer mortality (36). Guidelines from professional gastrointestinal organizations recommend early endoscopic examination for all patients over 55 with gastrointestinal symptoms and those presenting with alarm symptoms (42). However, in South Africa, the demand for endoscopy far exceeds the available resources (43). The limited success of tobacco control (44), along with resources allocated predominantly toward the management of communicable diseases, including AIDS and tuberculosis, which has resulted in the burden of noncommunicable diseases (NCDs) remaining unaddressed (45), may also contribute to the trends observed in South Africa.

In general, except for India, females tend to exhibit a more favorable profile than males in terms of age, period, and cohort effects, both globally and within the BRICS countries, which is consistent with previous research findings (46). This trend may be attributed to the protective effects of estrogen and gender differences in behavioral patterns, such as smoking and alcohol consumption (47, 48). In India, tobacco use is generally higher among males than females. Studies have shown that due to higher tobacco consumption among males, the incidence of cancer in males is higher than that in females in the Kashmir Valley region (49). On a biological level, the presence of estrogen receptor ER-*β* in gastric cancer may offer protective effects against invasiveness and lymphatic metastasis (50, 51). The use of anti-estrogen drugs may accelerate tumor progression or increase the overall risk of gastric adenocarcinoma (52, 53). Despite the higher prevalence of stomach cancer in males, the gender differences become negligible when comparing males to postmenopausal females. Furukawa et al. found that compared to untreated male rats, the incidence of stomach cancer was lower in female, castrated male, and estrogen-treated male rats, with histological differentiation also being lower (54). Physiological differences between genders may thus be a biological factor contributing to the higher gastric cancer mortality in males. Additionally, males may have a higher susceptibility to certain genetic mutations or differences in gastric environments, which could increase the incidence of stomach cancer. In India, among younger age groups (15–34 years), females experience higher mortality rates than males, possibly due to the significantly lower social status of females, which limits their access to healthcare resources and leads to concerning neglect of their health conditions (36).

The impact of the COVID-19 pandemic must also be considered. After 2019, the mortality rate from stomach cancer across countries showed a relatively steady trend, with mortality rates among middle-aged and older adult populations either stabilizing or declining. This could partly be attributed to the higher mortality rate among the older adult due to the pandemic, which consequently reduced the number of middle-aged and older adult individuals dying from gastric cancer. As BRICS countries are in a phase of rapid development, adopting a phased strategy to enhance stomach cancer prevention measures will be highly beneficial. Given the strong association between smoking, alcohol consumption, and the development of stomach cancer (55, 56), smoking cessation and alcohol reduction play a crucial role in both the prevention and treatment of gastric cancer. Public health education is essential to raise awareness and promote healthier behaviors. Moreover, foods rich in carcinogenic nitrosamines are major contributors to stomach cancer. In India, many dietary products in the Kashmir Valley are high in carcinogenic nitrosamines, resulting in a significantly higher incidence of stomach cancer compared to other cities in India (57). On the other hand, public knowledge and preventive health perceptions need to be strengthened, especially in economically underdeveloped rural areas. Studies have shown that nearly one-third of individuals do not recognize the early signs of the disease, with an average delay of more than 100 days from the onset of symptoms to the first consultation with a doctor. This often leads to missed opportunities for early diagnosis of serious conditions (58). Early screening for stomach cancer is critical, as patients diagnosed through screening tend to have a better prognosis than those diagnosed by other means (59). Relevant authorities should focus on improving public awareness of prevention strategies.

Based on the results of our Bayesian generalized linear model analysis, there is a significant association between the intake of PUFA, sodium, trans fats, and calcium and stomach cancer mortality in BRICS countries. Research indicates that excessive PUFA intake may be linked to an increased risk of gastric cancer (60). Moreover, sodium intake has long been recognized as a key risk factor for stomach cancer, particularly in Asia. High-salt diets are considered a major contributor to stomach cancer development, primarily through increased gastric mucosal damage and the promotion of *H. pylori* growth (61). In Brazil, a study highlighted the correlation between trans fat intake and stomach cancer, particularly in urban areas where dietary patterns are more Westernized, leading to increased trans fat consumption (62). This increase in trans fat intake may be a potential factor contributing to the rising stomach cancer mortality in BRICS countries.

Furthermore, calcium is considered a potential protective factor against stomach cancer. It is thought to reduce gastric acid secretion and enhance the mucosal barrier, thereby lowering cancer risk. Several studies have found a strong association between low calcium intake and stomach cancer, particularly in developing countries like India and South Africa, where calcium consumption tends to be lower (63). However, some research suggests that calcium’s protective effect may require the synergy of other micronutrients, such as vitamin D. Therefore, attention should be given to calcium intake in BRICS countries, especially in regions with low calcium consumption, where dietary adjustments could help improve stomach cancer prevention.

To the best of our knowledge, this study is the first to apply the APC model to analyze global stomach cancer mortality rates, particularly in BRICS countries. By incorporating age, period, and cohort effects, our research offers a refined understanding of mortality risk sources, which differs from the GBD 2019 publication (29). We also assess local variations in mortality rates, age distributions, and age at death globally and within BRICS nations. By incorporating age, period, and cohort effects, our research offers a refined understanding of mortality risk sources, differing from the GBD 2019 publication. We also assess local variations in mortality rates, age distributions, and age at death globally and within BRICS nations. This analysis yields critical insights into stomach cancer mortality trends and healthcare service effectiveness, providing a valuable resource for decision-makers and healthcare professionals. However, some limitations should be acknowledged. First, there is an absence of a more detailed subnational analysis in this study. Regional disparities in health outcomes and healthcare access were not fully captured, potentially overlooking significant variations in disease burden and healthcare resource distribution across different geographical areas within the country. Second, the use of five-year age intervals, while standard in APC modeling, may attenuate subtle variations in age, period, and cohort effects. Finally, GBD estimates are based on standardized Bayesian regression models, and in countries with limited diagnostic resources, this leads to wider confidence intervals due to lower detection rates of incidence.

## Conclusion

Despite observed reductions in stomach cancer mortality across the BRICS nations, these declines have exhibited considerable variability among the member countries. We acknowledge the diverse environmental contexts characteristic of the BRICS nations and recommend a phased strategy for the enhancement of stomach cancer prevention initiatives. Such efforts should be customized to the unique circumstances of each country, while effectively utilizing the available policy-driven resources, human capital, and financial support. Particularly for India and Brazil, measures such as strengthening foundational healthcare infrastructure, promoting disease prevention strategies, enhancing medical and healthcare services, and implementing comprehensive health promotion policies may further alleviate the substantial burden of stomach cancer mortality.

## Data Availability

The data analyzed in this study is subject to the following licenses/restrictions: the datasets generated during and/or analyzed during the current study are available from the corresponding author on reasonable request. Requests to access these datasets should be directed to Weihong Wang, wangweihong3001@163.com.

## Footnotes

^1^ https://ghdx.healthdata.org/gbd-2021
