# Supplementary material for: Time trends in stomach cancer mortality across the BRICS: an age-period-cohort analysis for the GBD 2021
Source: Front Public Health. 2025 Feb 28;13:1506925. doi: 10.3389/fpubh.2025.1506925 (PMC11906716; doi:10.3389/fpubh.2025.1506925)
Supplement: Supplementary file 1 [file Data_Sheet_1.docx]

**Supplementary Material**

**Time trends in stomach cancer mortality across the BRICS: an age-period-cohort analysis for the GBD 2021**

The supplementary material includes 1 supplementary tables.

**Supplementary Tables**

| Supplementary Table 1. Stomach cancer mortality rates estimated coefficients for the age, period and cohort effects in global and BRICS. | | | |
| --- | --- | --- | --- |
| Variables | Coefficients (95%CI) | | |
|  | Both | Female | Male |
| **Global** |  |  |  |
| Age |  |  |  |
| 15-19 | 0.43 (0.38, 0.49) | 0.44 (0.39, 0.5) | 0.42 (0.36, 0.5) |
| 20-24 | 0.97 (0.89, 1.06) | 1.07 (0.99, 1.15) | 0.87 (0.78, 0.98) |
| 25-29 | 1.86 (1.75, 1.98) | 2.03 (1.93, 2.14) | 1.7 (1.57, 1.85) |
| 30-34 | 3.85 (3.69, 4.02) | 3.59 (3.44, 3.73) | 4.09 (3.88, 4.31) |
| 35-39 | 6.71 (6.5, 6.94) | 5.56 (5.38, 5.74) | 7.8 (7.51, 8.11) |
| 40-44 | 11.09 (10.8, 11.38) | 7.97 (7.76, 8.19) | 14.02 (13.61, 14.44) |
| 45-49 | 16.16 (15.81, 16.52) | 10.16 (9.92, 10.41) | 21.85 (21.32, 22.4) |
| 50-54 | 24.91 (24.44, 25.38) | 14.48 (14.18, 14.79) | 34.98 (34.26, 35.72) |
| 55-59 | 34.46 (33.87, 35.06) | 19.07 (18.7, 19.44) | 49.77 (48.84, 50.72) |
| 60-64 | 45.24 (44.51, 45.99) | 25.1 (24.65, 25.57) | 65.96 (64.78, 67.17) |
| 65-69 | 57.2 (56.29, 58.13) | 32.3 (31.72, 32.88) | 83.94 (82.46, 85.44) |
| 70-74 | 71.65 (70.51, 72.81) | 41.4 (40.67, 42.13) | 106.03 (104.16, 107.95) |
| 75-79 | 81.91 (80.44, 83.42) | 50.26 (49.29, 51.24) | 120.4 (117.95, 122.89) |
| 80-84 | 90.07 (88.31, 91.87) | 58.04 (56.87, 59.23) | 132.8 (129.79, 135.87) |
| 85-89 | 99.94 (97.66, 102.28) | 65.43 (63.96, 66.93) | 152.54 (148.34, 156.86) |
| 90-94 | 107.77 (104.39, 111.25) | 75.45 (73.35, 77.61) | 160.61 (153.94, 167.58) |
| Period |  |  |  |
| 1984 | 1.52 (1.49, 1.55) | 1.58 (1.55, 1.61) | 1.49 (1.46, 1.53) |
| 1989 | 1.31 (1.29, 1.34) | 1.34 (1.32, 1.37) | 1.3 (1.28, 1.33) |
| 1994 | 1.14 (1.12, 1.16) | 1.15 (1.14, 1.17) | 1.14 (1.12, 1.16) |
| 1999 | 1 (1, 1) | 1 (1, 1) | 1 (1, 1) |
| 2004 | 0.93 (0.91, 0.94) | 0.9 (0.89, 0.92) | 0.94 (0.92, 0.96) |
| 2009 | 0.78 (0.77, 0.79) | 0.74 (0.73, 0.76) | 0.8 (0.78, 0.81) |
| 2014 | 0.68 (0.67, 0.69) | 0.64 (0.63, 0.65) | 0.7 (0.68, 0.71) |
| 2019 | 0.62 (0.61, 0.63) | 0.6 (0.59, 0.61) | 0.63 (0.62, 0.65) |
| Cohort |  |  |  |
| 1890--1894 | 2.34 (2.09, 2.62) | 2.94 (2.69, 3.21) | 2.01 (1.7, 2.38) |
| 1895-1899 | 2.39 (2.26, 2.53) | 3.05 (2.91, 3.19) | 2.06 (1.9, 2.23) |
| 1900-1904 | 2.33 (2.25, 2.41) | 2.89 (2.8, 2.99) | 2.1 (2.01, 2.2) |
| 1905-1909 | 2.21 (2.15, 2.27) | 2.69 (2.62, 2.76) | 2.05 (1.98, 2.11) |
| 1910-1914 | 2.09 (2.05, 2.14) | 2.49 (2.44, 2.55) | 1.98 (1.92, 2.03) |
| 1915-1919 | 1.97 (1.93, 2.01) | 2.29 (2.24, 2.33) | 1.87 (1.83, 1.92) |
| 1920-1924 | 1.8 (1.76, 1.83) | 2.05 (2.01, 2.09) | 1.73 (1.7, 1.77) |
| 1925-1929 | 1.69 (1.66, 1.72) | 1.86 (1.83, 1.9) | 1.64 (1.61, 1.67) |
| 1930-1934 | 1.54 (1.52, 1.57) | 1.64 (1.61, 1.67) | 1.51 (1.48, 1.54) |
| 1935-1939 | 1.35 (1.32, 1.37) | 1.39 (1.37, 1.42) | 1.33 (1.31, 1.36) |
| 1940-1944 | 1.14 (1.12, 1.16) | 1.16 (1.14, 1.19) | 1.13 (1.11, 1.15) |
| 1945-1949 | 1 (1, 1) | 1 (1, 1) | 1 (1, 1) |
| 1950-1954 | 0.87 (0.86, 0.89) | 0.87 (0.85, 0.89) | 0.88 (0.86, 0.9) |
| 1955-1959 | 0.73 (0.71, 0.75) | 0.72 (0.7, 0.74) | 0.74 (0.72, 0.76) |
| 1960-1964 | 0.62 (0.6, 0.63) | 0.61 (0.6, 0.63) | 0.62 (0.61, 0.64) |
| 1965-1969 | 0.56 (0.54, 0.57) | 0.54 (0.52, 0.56) | 0.57 (0.55, 0.59) |
| 1970-1974 | 0.47 (0.45, 0.48) | 0.46 (0.45, 0.48) | 0.47 (0.45, 0.49) |
| 1975-1979 | 0.39 (0.37, 0.41) | 0.39 (0.37, 0.41) | 0.39 (0.37, 0.42) |
| 1980-1984 | 0.34 (0.32, 0.36) | 0.34 (0.32, 0.36) | 0.34 (0.32, 0.37) |
| 1985-1989 | 0.31 (0.29, 0.35) | 0.3 (0.28, 0.33) | 0.33 (0.29, 0.37) |
| 1990-1994 | 0.27 (0.23, 0.31) | 0.26 (0.23, 0.29) | 0.28 (0.23, 0.34) |
| 1995-1999 | 0.22 (0.17, 0.28) | 0.22 (0.18, 0.27) | 0.22 (0.16, 0.31) |
| 2000-2004 | 0.18 (0.11, 0.28) | 0.18 (0.12, 0.27) | 0.17 (0.09, 0.32) |
| **Brazil** |  |  |  |
| Age |  |  |  |
| 15-19 | 0.25 (0.21， 0.3) | 0.19 (0.15, 0.24) | 0.3 (0.24, 0.38) |
| 20-24 | 0.57 (0.51, 0.63) | 0.48 (0.41, 0.56) | 0.62 (0.53, 0.73) |
| 25-29 | 1.31 (1.21, 1.41) | 1.08 (0.97, 1.21) | 1.47 (1.33, 1.63) |
| 30-34 | 2.67 (2.53, 2.82) | 2.06 (1.89, 2.24) | 3.21 (2.98, 3.45) |
| 35-39 | 4.53 (4.34, 4.72) | 3.27 (3.05, 3.5) | 5.74 (5.43, 6.06) |
| 40-44 | 7.9 (7.64, 8.17) | 5.19 (4.9, 5.51) | 10.65 (10.21, 11.11) |
| 45-49 | 12.66 (12.3, 13.02) | 7.49 (7.12, 7.89) | 18.06 (17.45, 18.7) |
| 50-54 | 19.46 (18.98, 19.95) | 10.54 (10.07, 11.04) | 29 (28.15, 29.87) |
| 55-59 | 27.37 (26.75, 28) | 14.46 (13.86 15.08) | 41.62 (40.5, 42.76) |
| 60-64 | 37.54 (36.74, 38.36) | 19.88 (19.1, 20.68) | 57.67 (56.21, 59.17) |
| 65-69 | 48.88 (47.86, 49.92) | 26.8 (25.79, 27.85) | 75.05 (73.19, 76.96) |
| 70-74 | 61.28 (60, 62.59) | 35.4 (34.07, 36.77) | 93.68 (91.34, 96.09) |
| 75-79 | 73.48 (71.75, 75.25) | 47.22 (45.27, 49.25) | 108.97 (105.85, 112.19) |
| 80-84 | 83.69 (81.57, 85.87) | 57.96 (55.46, 60.57) | 122.08 (118.23, 126.06) |
| 85-89 | 89.03 (86.43, 91.71) | 65.28 (62.19, 68.51) | 128.62 (123.76, 133.66) |
| 90-94 | 90.23 (86.73, 93.86) | 69.72 (65.68, 74.01) | 127.66 (120.84, 134.86) |
| Period |  |  |  |
| 1984 | 1.37 (1.33, 1.4) | 1.31 (1.25, 1.36) | 1.39 (1.35, 1.44) |
| 1989 | 1.21 (1.19, 1.24) | 1.17 (1.13, 1.22) | 1.23 (1.2, 1.27) |
| 1994 | 1.11 (1.08, 1.13) | 1.11 (1.07, 1.15) | 1.1 (1.07, 1.13) |
| 1999 | 1 (1, 1) | 1 (1 ,1) | 1( 1, 1) |
| 2004 | 0.91 (0.89, 0.93) | 0.91 (0.88, 0.94) | 0.91 (0.89, 0.93) |
| 2009 | 0.78 (0.76, 0.8) | 0.78 (0.75, 0.81) | 0.78 (0.76, 0.81) |
| 2014 | 0.7 (0.68, 0.71) | 0.71 (0.68, 0.73) | 0.7 (0.68, 0.72) |
| 2019 | 0.64 (0.63, 0.66) | 0.65 (0.62, 0.67) | 0.64 (0.62, 0.66) |
| Cohort |  |  |  |
| 1890--1894 | 3.46 (3.01, 3.99) | 3.77 (3.11, 4.57) | 3.23 (2.62, 3.99) |
| 1895-1899 | 3.13 (2.9, 3.37) | 3.44 (3.09, 3.84) | 2.84 (2.55, 3.16) |
| 1900-1904 | 3.14 (2.99, 3.29) | 3.5 (3.25, 3.76) | 2.8 (2.63, 2.98) |
| 1905-1909 | 2.74 (2.65, 2.84) | 3.02 (2.85, 3.2) | 2.49 (2.38, 2.61) |
| 1910-1914 | 2.36 (2.29, 2.43) | 2.54 (2.42, 2.68) | 2.18 (2.1, 2.27) |
| 1915-1919 | 2.1 (2.04, 2.15) | 2.22 (2.12, 2.33) | 1.98 (1.91, 2.05) |
| 1920-1924 | 1.84 (1.8, 1.89) | 1.89 (1.81, 1.98) | 1.78 (1.73, 1.84) |
| 1925-1929 | 1.66 (1.62, 1.7) | 1.7 (1.63, 1.78) | 1.63 (1.58, 1.68) |
| 1930-1934 | 1.49 (1.46, 1.53) | 1.5 (1.44, 1.57) | 1.48 (1.44, 1.53) |
| 1935-1939 | 1.32 (1.29, 1.35) | 1.32 (1.26, 1.37) | 1.32 (1.28, 1.36) |
| 1940-1944 | 1.16 (1.13, 1.19) | 1.15 (1.1, 1.2) | 1.16 (1.13, 1.19) |
| 1945-1949 | 1 (1 ,1) | 1( 1 ,1) | 1 (1 ,1) |
| 1950-1954 | 0.91 (0.89, 0.94) | 0.94 (0.9, 0.98) | 0.91 (0.88, 0.93) |
| 1955-1959 | 0.83 (0.8, 0.85) | 0.87 (0.83, 0.92) | 0.81 (0.79, 0.84) |
| 1960-1964 | 0.72 (0.7, 0.75) | 0.8 (0.76, 0.84) | 0.69 (0.67, 0.72) |
| 1965-1969 | 0.65 (0.62, 0.67) | 0.74 (0.7, 0.79) | 0.6 (0.58, 0.63) |
| 1970-1974 | 0.59 (0.56, 0.62) | 0.7 (0.65, 0.76) | 0.54 (0.5, 0.57) |
| 1975-1979 | 0.55 (0.51, 0.58) | 0.67 (0.61, 0.74) | 0.48 (0.45, 0.52) |
| 1980-1984 | 0.51 (0.47, 0.55) | 0.61 (0.54, 0.69) | 0.45 (0.41, 0.5) |
| 1985-1989 | 0.47 (0.42, 0.52) | 0.56 (0.47, 0.65) | 0.42 (0.36, 0.49) |
| 1990-1994 | 0.44 (0.37, 0.52) | 0.49 (0.38, 0.63) | 0.42 (0.33, 0.53) |
| 1995-1999 | 0.44 (0.34, 0.57) | 0.49 (0.33, 0.72) | 0.42 (0.29, 0.6) |
| 2000-2004 | 0.38 (0.22, 0.64) | 0.45 (0.21, 1) | 0.34 (0.17, 0.7) |
| **China** |  |  |  |
| Age |  |  |  |
| 15-19 | 0.99 (0.78, 1.24) | 1.06 (0.86, 1.3) | 0.99 (0.75, 1.32) |
| 20-24 | 2.09 (1.79, 2.43) | 2.31 (2.02, 2.64) | 2.04 (1.68, 2.48) |
| 25-29 | 3.74 (3.36, 4.17) | 4.18 (3.8, 4.59) | 3.61 (3.14, 4.15) |
| 30-34 | 8.26 (7.69, 8.87) | 7.39 (6.91, 7.91) | 9.27 (8.51, 10.09) |
| 35-39 | 15.08 (14.31, 15.89) | 11.55 (10.96, 12.17) | 18.43 (17.36, 19.57) |
| 40-44 | 25.28 (24.27, 26.33) | 16.47 (15.77, 17.2) | 33.28 (31.79, 34.84) |
| 45-49 | 35.12 (33.91, 36.37) | 18.89 (18.16, 19.64) | 49.78 (47.9, 51.72) |
| 50-54 | 53.74 (52.18, 55.36) | 27.79 (26.88, 28.73) | 77.64 (75.16, 80.21) |
| 55-59 | 73.51 (71.55, 75.52) | 36.86 (35.76, 38) | 107.83 (104.68, 111.07) |
| 60-64 | 93.89 (91.5, 96.34) | 47.55 (46.2, 48.94) | 138.30 (134.43, 142.27) |
| 65-69 | 116.87 (113.94, 119.87) | 60.35 (58.68, 62.07) | 173.07 (168.29, 177.99) |
| 70-74 | 151.00 (147.24, 154.86) | 78.68 (76.56, 80.87) | 226.61 (220.34, 233.07) |
| 75-79 | 170.50 (165.62, 175.53) | 91.83 (89.05, 94.69) | 258.72 (250.36, 267.35) |
| 80-84 | 180.60 (174.75, 186.63) | 100.86 (97.57, 104.27) | 281.15 (270.6, 292.1) |
| 85-89 | 207.22 (198.79, 216.01) | 105.64 (101.49, 109.95) | 377.22 (358.97, 396.39) |
| 90-94 | 220.44 (205.98, 235.91) | 113.77 (107.31, 120.61) | 468.68 (429.4, 511.55) |
| Period |  |  |  |
| 1984 | 1.56 (1.5, 1.61) | 1.69 (1.64, 1.75) | 1.50 (1.44, 1.56) |
| 1989 | 1.34 (1.3, 1.38) | 1.41 (1.37, 1.45) | 1.31 (1.27, 1.36) |
| 1994 | 1.13 (1.1 1.16) | 1.17 (1.14, 1.2) | 1.12 (1.09, 1.15) |
| 1999 | 1.00 (1, 1) | 1.00 (1, 1) | 1.00 (1, 1) |
| 2004 | 0.99 (0.97, 1.02) | 0.94 (0.92, 0.96) | 1.02 (0.99, 1.05) |
| 2009 | 0.78（0.75,0.8) | 0.69 (0.67, 0.71) | 0.82 (0.79, 0.84) |
| 2014 | 0.64（0.62， 0.66) | 0.54 (0.52, 0.56) | 0.69 (0.67, 0.72) |
| 2019 | 0.58（0.56, 0.6) | 0.51 (0.49, 0.53) | 0.61 (0.59, 0.64) |
| Cohort |  |  |  |
| 1890--1894 | 2.47 (1.82, 3.37) | 3.57 (2.8, 4.53) | 2.00 (1.3, 3.07) |
| 1895-1899 | 2.30 (2, 2.65) | 3.39 (3.03, 3.8) | 1.93 (1.59, 2.33) |
| 1900-1904 | 2.21 (2.05, 2.39) | 3.09 (2.89, 3.3) | 1.96 (1.77, 2.17) |
| 1905-1909 | 2.19 (2.08 2.3) | 2.96 (2.82, 3.1) | 1.98 (1.86, 2.11) |
| 1910-1914 | 2.09 (2, 2.17) | 2.73 (2.63, 2.84) | 1.91 (1.82, 2) |
| 1915-1919 | 2.02 (1.95, 2.09) | 2.56 (2.47, 2.65) | 1.88 (1.8, 1.95) |
| 1920-1924 | 1.88 (1.82, 1.94) | 2.33 (2.26, 2.41) | 1.75 (1.69, 1.81) |
| 1925-1929 | 1.73 (1.68, 1.78) | 2.08 (2.01, 2.14) | 1.62 (1.57, 1.67) |
| 1930-1934 | 1.55 (1.51, 1.6) | 1.78 (1.73, 1.83) | 1.47 (1.42, 1.51) |
| 1935-1939 | 1.36 (1.32, 1.4) | 1.49 (1.45, 1.54) | 1.30 (1.26, 1.34) |
| 1940-1944 | 1.15 (1.12, 1.19) | 1.21 (1.17, 1.25) | 1.13 (1.09, 1.16) |
| 1945-1949 | 1.00 (1, 1) | 1.00 (1, 1) | 1.00 (1, 1) |
| 1950-1954 | 0.81 (0.78, 0.83) | 0.78 (0.75, 0.81) | 0.83 (0.8, 0.85) |
| 1955-1959 | 0.67 (0.65, 0.7) | 0.62 (0.6, 0.64) | 0.70 (0.67, 0.73) |
| 1960-1964 | 0.54 (0.52, 0.56) | 0.48 (0.46, 0.51) | 0.57 (0.55, 0.6) |
| 1965-1969 | 0.47 (0.45, 0.5) | 0.41 (0.39, 0.43) | 0.51 (0.48, 0.54) |
| 1970-1974 | 0.39 (0.36, 0.41) | 0.33 (0.31, 0.35) | 0.42 (0.39, 0.45) |
| 1975-1979 | 0.34 (0.31, 0.37) | 0.28 (0.25, 0.3) | 0.38 (0.34, 0.42) |
| 1980-1984 | 0.31 (0.27, 0.35) | 0.24 (0.21, 0.27) | 0.35 (0.3, 0.4) |
| 1985-1989 | 0.28 (0.23, 0.33) | 0.21 (0.18, 0.25) | 0.33 (0.27, 0.4) |
| 1990-1994 | 0.24 (0.18, 0.32) | 0.18 (0.14, 0.23) | 0.28 (0.2, 0.4) |
| 1995-1999 | 0.19 (0.11, 0.33) | 0.14 (0.09, 0.23) | 0.23 (0.12, 0.44) |
| 2000-2004 | 0.13 (0.04, 0.43) | 0.10 (0.03, 0.29) | 0.15 (0.04, 0.64) |
| **India** |  |  |  |
| Age |  |  |  |
| 15-19 | 0.12 (0.09, 0.18) | 0.17 (0.13, 0.23) | 0.08 (0.05, 0.14) |
| 20-24 | 0.41 (0.33, 0.5) | 0.61 (0.51, 0.72) | 0.22 (0.16, 0.31) |
| 25-29 | 0.75 (0.64, 0.87) | 1.01 (0.89, 1.15) | 0.49 (0.4, 0.61) |
| 30-34 | 1.53 (1.37, 1.71) | 1.73 (1.56, 1.92) | 1.32 (1.15, 1.52) |
| 35-39 | 2.75 (2.52, 2.99) | 2.65 (2.44, 2.89) | 2.8 (2.53, 3.09) |
| 40-44 | 4.6 (4.29, 4.93) | 3.79 (3.51, 4.08) | 5.27 (4.88, 5.7) |
| 45-49 | 7.07 (6.65, 7.51) | 5.22 (4.88, 5.59) | 8.64 (8.1, 9.22) |
| 50-54 | 11.47 (10.88, 12.1) | 7.64 (7.17, 8.13) | 14.87 (14.06, 15.73) |
| 55-59 | 17.2 (16.37, 18.06) | 10.74 (10.13, 11.39) | 23.28 (22.11, 24.51) |
| 60-64 | 23.59 (22.53, 24.69) | 14.98 (14.19, 15.82) | 32.17 (30.66, 33.76) |
| 65-69 | 30.7 (29.34, 32.13) | 18.44 (17.47, 19.46) | 43.33 (41.33, 45.44) |
| 70-74 | 34.71 (33.13, 36.38) | 21.84 (20.67, 23.08) | 48.63 (46.29, 51.09) |
| 75-79 | 34.84 (32.85, 36.95) | 24.16 (22.59, 25.83) | 47.2 (44.32, 50.26) |
| 80-84 | 42.74 (39.96, 45.72) | 30.58 (28.36, 32.97) | 57.92 (53.84, 62.31) |
| 85-89 | 51.6 (47.31, 56.27) | 35.85 (32.56, 39.47) | 72.77 (66.22, 79.98) |
| 90-94 | 56.97 (49.64, 65.38) | 40.01 (34.53, 46.35) | 82.21 (70.54, 95.82) |
| Period |  |  |  |
| 1984 | 1.34 (1.26, 1.42) | 1.38 (1.29, 1.47) | 1.31 (1.23, 1.4) |
| 1989 | 1.21 (1.14, 1.28) | 1.23 (1.16, 1.31) | 1.19 (1.12, 1.26) |
| 1994 | 1.1 (1.04, 1.15) | 1.09 (1.03, 1.16) | 1.09 (1.03, 1.15) |
| 1999 | 1 (1, 1) | 1 (1, 1) | 1 (1, 1) |
| 2004 | 0.88 (0.84, 0.93) | 0.86 (0.81, 0.91) | 0.9 (0.85, 0.95) |
| 2009 | 0.89 (0.84, 0.93) | 0.87 (0.82, 0.92) | 0.91 (0.86, 0.96) |
| 2014 | 0.88 (0.84, 0.93) | 0.9 (0.85, 0.95) | 0.89 (0.84, 0.95) |
| 2019 | 0.85 (0.81, 0.9) | 0.89 (0.84, 0.94) | 0.85( 0.8, 0.9) |
| Cohort |  |  |  |
| 1890--1894 | 1.08 (0.53, 2.19) | 1.12 (0.53, 2.36) | 0.97 (0.44, 2.14) |
| 1895-1899 | 1.11 (0.8, 1.54) | 1.17 (0.83, 1.66) | 1.01 (0.7, 1.45) |
| 1900-1904 | 1.14 (0.95, 1.37) | 1.16 (0.9, 1.42) | 1.07 (0.87, 1.31) |
| 1905-1909 | 1.21 (1.08, 1.37) | 1.2 (1.05, 1.37) | 1.17 (1.03, 1.33) |
| 1910-1914 | 1.24 (1.13, 1.36) | 1.22 (1.1, 1.36) | 1.21 (1.1, 1.34) |
| 1915-1919 | 1.26 (1.17, 1.36) | 1.24 (1.14, 1.36) | 1.25 (1.15, 1.35) |
| 1920-1924 | 1.31 (1.24, 1.4) | 1.3 (1.2, 1.39) | 1.29 (1.21, 1.38) |
| 1925-1929 | 1.31 (1.24, 1.39) | 1.3 (1.21, 1.38) | 1.29 (1.22, 1.37) |
| 1930-1934 | 1.29 (1.23, 1.36) | 1.28 (1.21, 1.37) | 1.28 (1.21, 1.36) |
| 1935-1939 | 1.18 (1.12, 1.24) | 1.18 (1.11, 1.25) | 1.19 (1.12, 1.25) |
| 1940-1944 | 1.11 (1.06, 1.17) | 1.12 (1.06, 1.19) | 1.11 (1.05,1.17) |
| 1945-1949 | 1 (1, 1) | 1 (1, 1) | 1 (1, 1) |
| 1950-1954 | 0.92 (0.87, 0.97) | 0.92 (0.86, 0.98) | 0.93 (0.88, 0.99) |
| 1955-1959 | 0.86 (0.81, 0.91) | 0.85 (0.79, 0.91) | 0.87 (0.82, 0.93) |
| 1960-1964 | 0.84 (0.78, 0.89) | 0.86 (0.8, 0.93) | 0.84 (0.78, 0.9) |
| 1965-1969 | 0.7 (0.65, 0.76) | 0.71 (0.65, 0.77) | 0.72 (0.66, 0.78) |
| 1970-1974 | 0.69 (0.63, 0.75) | 0.7 (0.64, 0.77) | 0.69 (0.62, 0.77) |
| 1975-1979 | 0.62 (0.55, 0.7) | 0.62 (0.55, 0.69) | 0.63 (0.55, 0.73) |
| 1980-1984 | 0.52 (0.44, 0.6) | 0.52 (0.45, 0.6) | 0.52 (0.42, 0.63) |
| 1985-1989 | 0.45 (0.36, 0.56) | 0.45 (0.37, 0.54) | 0.46( 0.34, 0.61) |
| 1990-1994 | 0.37 (0.27, 0.52) | 0.37 (0.28, 0.48) | 0.38 (0.23, 0.62) |
| 1995-1999 | 0.29 (0.17, 0.5) | 0.27 (0.17, 0.42) | 0.34 (0.15, 0.75) |
| 2000-2004 | 0.22 (0.07, 0.76) | 0.2 (0.07, 0.57) | 0.25 (0.04, 1.42) |
| **Russian Federation** |  |  |  |
| Age |  |  |  |
| 15-19 | 0.59 (0.36, 0.95) | 0.51 (0.26, 0.99) | 0.64 (0.43, 0.96) |
| 20-24 | 1.44 (1.09, 1.89) | 1.34 (0.92, 1.93) | 1.46 (1.16, 1.84) |
| 25-29 | 3.12 (2.63,3.7) | 2.89 (2.29, 3.65) | 3.19 (2.77, 3.67) |
| 30-34 | 6.45 (5.77, 7.21) | 5.67 (4.84, 6.65) | 6.99 (6.4, 7.64) |
| 35-39 | 11.25 (10.37, 12.19) | 8.81 (7.79, 9.95) | 13.51 (12.7, 14.36) |
| 40-44 | 18.13 (17.02, 19.31) | 12.21 (11.03, 13.5) | 24.1 (23.01, 25.24) |
| 45-49 | 26.77 (25.44, 28.180) | 15.76 (14.47, 17.17) | 38.5 (37.11, 39.93) |
| 50-54 | 38.49 (36.8, 40.26) | 20.25 (18.76, 21.85) | 59.32 (57.46, 61.24) |
| 55-59 | 50.09 (48.06, 52.21) | 25.47 (23.76, 27.31) | 80.98 (78.62, 83.41) |
| 60-64 | 63.4 (60.89, 66.02) | 33.68 (31.5, 36) | 105.03 (102.01,108.14) |
| 65-69 | 73.71 (70.8, 76.74) | 42.73 (40.03, 45.61) | 122.38 (118.82, 126.04) |
| 70-74 | 80.03 (76.81, 83.4) | 50.67 (47.47, 54.08) | 132.47 (128.46, 136.61) |
| 75-79 | 83.21 (79.54, 87.05) | 57.45 (53.62, 61.55) | 134.28 (129.64, 139.08) |
| 80-84 | 77.09 (73.35, 81.02) | 55.84 (51.91, 60.07) | 125.67 (120.58, 130.97) |
| 85-89 | 60.11 (56.31, 64.16) | 45.67 (41.87, 49.82) | 93.54 (87.93, 99.52) |
| 90-94 | 61.82 (55.88, 68.39) | 48.87 (43.28, 55.17) | 88.5 (79.32, 98.74) |
| Period |  |  |  |
| 1984 | 1.81 (1.72, 1.9) | 1.89 (1.77, 2.03) | 1.85 (1.77, 1.92) |
| 1989 | 1.35 (1.3, 1.41) | 1.42 (1.33, 1.5) | 1.35 (1.3, 1.39) |
| 1994 | 1.26 (1.22, 1.31) | 1.25 (1.19, 1.31) | 1.28 (1.25, 1.32) |
| 1999 | 1 (1, 1) | 1 (1, 1） | 1 (1, 1) |
| 2004 | 0.85 (0.82, 0.88) | 0.86 (0.81, 0.9) | 0.85 (0.82, 0.87) |
| 2009 | 0.71 (0.68, 0.75) | 0.74 (0.7, 0.79) | 0.7 (0.67, 0.72) |
| 2014 | 0.62 (0.58, 0.65) | 0.64 (0.6, 0.69) | 0.6 (0.57, 0.62) |
| 2019 | 0.55 (0.52, 0.59) | 0.57 (0.52, 0.62) | 0.54 (0.51, 0.56) |
| Cohort |  |  |  |
| 1890--1894 | 3.08 (2.25, 4.2) | 3.43 (2.43, 4.84) | 3.25 (2.26, 4.68) |
| 1895-1899 | 3.06 (2.61, 3.57) | 3.38 (2.82, 4.06) | 3.28 (2.77, 3.88) |
| 1900-1904 | 3.01 (2.76, 3.29) | 3.42 (3.07, 3.82) | 3.19 (2.92, 3.48) |
| 1905-1909 | 3.06 (2.88, 3.26) | 3.46 (3.18, 3.78) | 3.14 (2.98, 3.32) |
| 1910-1914 | 2.94 (2.79, 3.1) | 3.38 (3.14, 3.65) | 2.98 (2.86, 3.11) |
| 1915-1919 | 2.56 (2.44, 2.69) | 3.01 (2.79, 3.24) | 2.57 (2.47, 2.67) |
| 1920-1924 | 2.28 (2.17, 2.38) | 2.71 (2.53, 2.91) | 2.31 (2.23, 2.4) |
| 1925-1929 | 2.09 (2.01, 2.18) | 2.33 (2.18, 2.49) | 2.07 (2, 2.13) |
| 1930-1934 | 1.87 (1.79, 1.95) | 2 (1.87, 2.15) | 1.82 (1.76, 1.88) |
| 1935-1939 | 1.54 (1.47, 1.6) | 1.57 (1.47, 1.69) | 1.54 (1.5, 1.59) |
| 1940-1944 | 1.34 (1.28, 1.4) | 1.38 (1.28, 1.49) | 1.35 (1.3, 1.39) |
| 1945-1949 | 1 (1, 1) | 1 (1, 1) | 1 (1, 1) |
| 1950-1954 | 0.86 (0.82, 0.9） | 0.87 (0.81, 0.95） | 0.86 (0.83, 0.89） |
| 1955-1959 | 0.68 (0.64, 0.72） | 0.71 (0.65, 0.77） | 0.67 (0.64, 0.7） |
| 1960-1964 | 0.55 (0.51, 0.59） | 0.6 (0.54, 0.67） | 0.53 (0.5, 0.55） |
| 1965-1969 | 0.44 (0.4, 0.48） | 0.5 (0.44, 0.58） | 0.42 (0.39, 0.44） |
| 1970-1974 | 0.37 (0.33, 0.42） | 0.43 (0.36, 0.51） | 0.34 (0.31, 0.37） |
| 1975-1979 | 0.34 (0.3, 0.4） | 0.39 (0.31, 0.48） | 0.32 (0.29, 0.36） |
| 1980-1984 | 0.28 (0.23, 0.35） | 0.32 (0.24, 0.42） | 0.27 (0.23, 0.31） |
| 1985-1989 | 0.22 (0.16, 0.3） | 0.25 (0.16, 0.37） | 0.21 (0.16, 0.27） |
| 1990-1994 | 0.19 (0.11, 0.33） | 0.22 (0.1, 0.46） | 0.16 (0.1, 0.27） |
| 1995-1999 | 0.14 (0.04, 0.45） | 0.16 (0.03, 0.77） | 0.12 (0.04, 0.33） |
| 2000-2004 | 0.12 (0.01, 1.19） | 0.14 (0.01, 3.15） | 0.11 (0.02, 0.72） |
| **South Africa** |  |  |  |
| Age |  |  |  |
| 15-19 | 0.15 (0.09, 0.25) | 0.16 (0.07, 0.35) | 0.13 (0.07, 0.25) |
| 20-24 | 0.43 (0.32, 0.58) | 0.54 (0.36, 0.81) | 0.32 (0.21, 0.49) |
| 25-29 | 0.95 (0.78, 1.15) | 1.11 (0.83, 1.47) | 0.79 (0.61, 1.03) |
| 30-34 | 2.13 (1.85, 2.46) | 2.07 (1.66, 2.58) | 2.17 (1.82, 2.58) |
| 35-39 | 3.62 (3.24, 4.05) | 2.94 (2.45, 3.53) | 4.23 (3.72, 4.82) |
| 40-44 | 5.17 (4.69, 5.7) | 3.45 (2.91, 4.09) | 6.89 (6.18, 7.68) |
| 45-49 | 7.36 (6.74, 8.03) | 4.32 (3.67, 5.07) | 10.69 (9.71, 11.77) |
| 50-54 | 12.43 (11.51, 13.42) | 6.57 (5.68, 7.59) | 19.05 (17.53, 20.7) |
| 55-59 | 17.78 (16.56, 19.09) | 9.31 (8.15, 10.65) | 27.73 (25.67, 29.96) |
| 60-64 | 22.49 (20.99, 24.09) | 13.29 (11.75, 15.04) | 34.24 (31.71, 36.97) |
| 65-69 | 30.28 (28.27, 32.44) | 18.51 (16.41, 20.89) | 46.61 (43.13, 50.37) |
| 70-74 | 37.18 (34.6, 39.96) | 23.77 (21.02, 26.89) | 56.99 (52.47, 61.9) |
| 75-79 | 47.98 (44.18, 52.12) | 34.96 (30.49, 40.09) | 68.25 (61.88, 75.28) |
| 80-84 | 72.4 (66.42, 78.91) | 58.77 (51.17, 67.51) | 94.15 (84.55, 104.83) |
| 85-89 | 93.51 (84.61, 103.33) | 77.72 (66.66, 90.61) | 121.24 (106.29, 138.29) |
| 90-94 | 108.8 94.25 125.61) | 98.66 80.81 120.45) | 126.17 101.76 156.45) |
| Period |  |  |  |
| 1984 | 1.19 (1.1, 1.29) | 1.39 (1.22, 1.58) | 1.04 (0.95, 1.14) |
| 1989 | 0.96 (0.89, 1.04) | 1.08 (0.95, 1.22) | 0.88 (0.8, 0.96) |
| 1994 | 0.91 (0.85, 0.98) | 0.95 (0.85, 1.07) | 0.87 (0.8, 0.94) |
| 1999 | 1 (1, 1) | 1 (1, 1) | 1 (1, 1) |
| 2004 | 0.94 (0.88, 1) | 0.97 (0.87, 1.08) | 0.93 (0.86, 1) |
| 2009 | 0.85 (0.8, 0.91) | 0.87 (0.78, 0.97) | 0.84 (0.78, 0.91) |
| 2014 | 0.74 (0.69, 0.8) | 0.78 (0.7, 0.88) | 0.72 (0.67, 0.78) |
| 2019 | 0.65 (0.6, 0.7) | 0.67 (0.6, 0.76) | 0.63 (0.58, 0.68) |
| Cohort |  |  |  |
| 1890--1894 | 1.12 (0.72, 1.75) | 1.07 (0.59, 1.93) | 1.2 (0.63, 2.28) |
| 1895-1899 | 1.2 (0.96, 1.51) | 1.19 (0.86, 1.63) | 1.22 (0.89, 1.66) |
| 1900-1904 | 1.25 (1.07, 1.46) | 1.28 (1.02, 1.6) | 1.23 (1, 1.51) |
| 1905-1909 | 1.26 (1.11, 1.43) | 1.32 (1.09, 1.6) | 1.22 (1.04, 1.43) |
| 1910-1914 | 1.18 (1.06, 1.32) | 1.28 (1.07, 1.52) | 1.13 (0.98, 1.3) |
| 1915-1919 | 1.1 (0.99, 1.21) | 1.19 (1.01, 1.4) | 1.05 (0.93, 1.18) |
| 1920-1924 | 1.1 (1.01, 1.2) | 1.18 (1.02, 1.37) | 1.06 (0.95, 1.17) |
| 1925-1929 | 1.07 (0.98, 1.16) | 1.13 (0.98, 1.3) | 1.05 (0.96, 1.16) |
| 1930-1934 | 1.05 (0.97, 1.14) | 1.13 (0.99, 1.29) | 1.04 (0.95, 1.13) |
| 1935-1939 | 1.04 (0.96, 1.12) | 1.1 (0.96, 1.25) | 1.04 (0.95, 1.13) |
| 1940-1944 | 1.06 (0.99, 1.14) | 1.1 (0.97, 1.26) | 1.04 (0.96, 1.13) |
| 1945-1949 | 1 (1, 1) | 1 (1, 1) | 1 (1, 1) |
| 1950-1954 | 0.96 (0.89, 1.04) | 0.94 (0.82, 1.08) | 0.97 (0.89, 1.06) |
| 1955-1959 | 0.86 (0.79, 0.94) | 0.86 (0.74, 1) | 0.88 (0.8, 0.96) |
| 1960-1964 | 0.79 (0.72, 0.86) | 0.83 (0.7, 0.98) | 0.79 (0.71, 0.88) |
| 1965-1969 | 0.72 (0.65, 0.8) | 0.78 (0.65, 0.94) | 0.71 (0.62, 0.8) |
| 1970-1974 | 0.66 (0.58, 0.75) | 0.69 (0.55, 0.85) | 0.66 (0.57, 0.77) |
| 1975-1979 | 0.54 (0.45, 0.63) | 0.51 (0.39, 0.67) | 0.57 (0.47, 0.69) |
| 1980-1984 | 0.39 (0.32, 0.49) | 0.35 (0.24, 0.5) | 0.45 (0.34, 0.58) |
| 1985-1989 | 0.32 (0.23, 0.44) | 0.25 (0.15, 0.42) | 0.41 (0.28, 0.59) |
| 1990-1994 | 0.27 (0.16, 0.46) | 0.18 (0.08, 0.42) | 0.42 (0.23, 0.77) |
| 1995-1999 | 0.28 (0.12, 0.66) | 0.17 (0.04, 0.74) | 0.45 (0.16, 1.22) |
| 2000-2004 | 0.29 (0.05, 1.59) | 0.19 (0.01, 3.49) | 0.41 (0.06, 2.95) |
